# Evolution of Radiation Therapy in Pancreas Cancer Management toward MRI-Guided Adaptive Radiation Therapy

**DOI:** 10.3390/jcm11185380

**Published:** 2022-09-13

**Authors:** Amulya Yalamanchili, Tarita O. Thomas, Salah Dajani, John P. Hayes

**Affiliations:** Northwestern Medicine, Department of Radiation Oncology, Chicago, IL 60611, USA

**Keywords:** locally advanced pancreas cancer, pancreas cancer, SBRT, radiation, MR-guided adaptive radiation, biological effective dose

## Abstract

Pancreas cancer has a poor prognosis despite aggressive treatment and is the fourth leading cause of cancer death in the United States. At diagnosis, most patients have either metastatic or locally advanced disease. In this article, we review the evolution of treatments in locally advanced pancreas cancer (LAPC) and discuss the various radiation therapy fractionation schemes. Furthermore, we examine the data supporting dose escalation and the delivery of ablative biologically effective doses in the setting of LAPC. Finally, we review the role of MRI-guided radiation therapy in escalating dose while sparing organs at risk in the era of stereotactic magnetic resonance-guided adaptive radiation therapy.

## 1. Introduction

Pancreas cancer is a challenging diagnosis with a poor prognosis despite aggressive treatment and is the fourth leading cause of cancer mortality in the United States [1,2]. Non-metastatic pancreatic cancer can be classified as resectable, borderline resectable, and unresectable or locally advanced disease. Locally advanced pancreatic cancer makes up approximately one-third of new pancreatic adenocarcinoma diagnoses. The five-year survival for localized, locally advanced, and distant metastatic disease is 9.0–31.7%, 1.9–8.7%, and 0.5%, respectively [3]. Currently, surgical resection is the only curative option for pancreatic cancer, yet the disease may be unresectable due involvement of nearby blood vessels. The unresectable disease can be treated with chemotherapy, radiation, or a combination approach. Radiation can be delivered in the palliative or definitive setting for locally advanced disease and has also been utilized neoadjuvantly for borderline resectable disease for downstaging purposes. While distant metastasis remains the predominant cause of disease progression, achieving local control is also important. This is illustrated by a postmortem study from Johns Hopkins University which showed that 30% of deaths in pancreatic cancer patients were due to locally advanced disease [4]. Radiotherapy improves local control and survival in patients with unresectable disease [5]. MRI-guided adaptive radiation therapy preserves these control and survival benefits while also reducing potential toxicities of radiotherapy. Optimal selection and timing of treatment modalities including radiotherapy are vital to patient outcomes in pancreatic cancer.

## 2. Methods

We used Google Scholar and PubMed to find relevant manuscripts. We restricted our search to papers published in English. We used the following terms in different combinations: “pancreas cancer”, “pancreatic cancer”, “unresectable”, “locally advanced”, “stereotactic”, “SBRT”, “CTgRT”, “MRI”, “MRgRT“, “radiotherapy”, “radiation”, “ablative”. Additional literature was also sought from references of included reviews and studies. A final shortlist of studies was selected based on relevance, particularly in those that investigated the outcomes of SBRT, CTgRT, and MRgRT in patients with inoperable locally advanced pancreas cancer.

## 3. Role of Radiotherapy

The role of combined chemotherapy and radiation in unresectable disease is controversial. Trials by the Gastrointestinal Tumor Study Group (GITSG) in the 1980s supported the use of radiation and concurrent chemotherapy over radiation or chemotherapy alone for unresectable disease. GITSG 9273 established the role for chemoradiation by demonstrating improved survival benefits with 5-fluorouracil (5-FU) and concurrent radiotherapy compared to radiotherapy alone in locally advanced pancreatic cancer. The median survival time from diagnosis was 10 months with 5-FU and radiation versus 5.5 months with radiation alone [6]. The follow-up study GITSG 9283 demonstrated improved median survival of 42 weeks with 5-FU and concurrent radiotherapy compared to 32 weeks with chemotherapy alone [7].

More recent trials demonstrate mixed results comparing chemoradiation to chemotherapy alone. A phase III French trial compared induction chemoradiation (5-FU and cisplatin with concurrent radiotherapy) followed by maintenance gemcitabine to gemcitabine alone. The chemoradiation arm resulted in increased toxicity and decreased effectiveness. Patients in the chemoradiation arm had a median survival of 8.6 months and a one-year survival of 32%, while patients in the gemcitabine alone arm had a median survival of 13 months and a one-year survival of 53%. Radiation was prescribed to 60 Gy to a large field, with a clinical target volume (CTV) including peripancreatic lymph nodes and celiac and hepatic hilar regions, and a 2 cm expansion to the planning target volume (PTV). Of note, due to the side effects of the chemoradiation regimen, only 83% received at least three-quarters of the prescribed radiation, and only 42% received at least three-quarters of the planned radiation and chemotherapy. Additionally, the number of gemcitabine infusions and median total dose of gemcitabine in the maintenance phase were significantly higher in the chemotherapy alone arm [8].

An Eastern Cooperative Oncology Group study (ECOG 4201) evaluated gemcitabine-based chemoradiation versus gemcitabine alone. Although this study closed early due to low accrual, median survival was improved in the chemoradiation arm, 11.1 versus 9.2 months [9].

LAP07, a phase III trial, showed no significant overall survival difference with chemoradiation compared to chemotherapy alone at a median follow-up of 36.7 months. Patients first received induction chemotherapy with either gemcitabine or gemcitabine-erlotinib. Patients with good tumor control then received either maintenance chemotherapy with gemcitabine or erlotinib, or radiation using 54 Gy in 1.8 Gy per fraction with concurrent capecitabine. The median survival was 16.5 months in the chemotherapy arm compared to 15.2 months in the chemoradiation arm. However, patients in the chemoradiation group had significantly decreased local progression, 32% versus 46% [10]. There was no increase in grade 3–4 toxicity except nausea.

The French phase III trial ECOG 4201 and LAP07 all utilized a three-dimensional conformal radiation therapy (3D-CRT) approach using conventional fractionation up to 60 Gy with 1.8–2 Gy per daily fractions over several weeks. Together, these conventionally fractionated chemoradiation trials using 3D-CRT showed a small local control benefit but minimal to no impact on survival.

## 4. Single Fraction Stereotactic Body Radiation Therapy

Conventionally fractionated radiotherapy requires five to six weeks of daily treatments, a significant portion of a patient’s limited life expectancy. It also offers relatively modest and inconsistent benefits in survival with a corresponding risk of significant side effects. Because of this, interest arose in a redesign of the targeting paradigm, notably coinciding with advances in image-guided radiation therapy technologies, with the hope that greater biologically effective doses could lead to better outcomes. Stereotactic body radiation therapy (SBRT), also known as stereotactic ablative radiation therapy (SABR), was the result.

Increasingly, SBRT is being utilized to definitively treat unresectable pancreatic cancer. SBRT delivers higher doses of radiation to the gross tumor volume in fewer fractions compared to conventional fractionation and does not include elective nodal treatment. This technique has been shown to improve local control as well as survival while minimizing interruption of systemic therapy. However, the SBRT prescription dose remains constrained by normal tissue toxicity, particularly in the gastrointestinal (GI) tract. Organs such as duodenum, jejunum, and stomach are radiosensitive, and ablative doses to these organs with serial functional subunits can be detrimental to organ function.

Early studies with single fraction SBRT had significant early and late GI toxicities. The safety of single fraction SBRT was evaluated in a phase I dose escalation study at Stanford where Koong et al. evaluated prescription doses of 15 Gy, 20 Gy, and 25 Gy in a single fraction. All six patients treated to 25 Gy had local control until the last follow-up or death, and the site of the first progression was distant. The median overall survival for patients treated to 25 Gy was 8 months with a median follow-up of 4.5 months. The duodenum received the highest dose of all surrounding organs at risk (OAR) due to its proximity to the pancreas. The mean doses of 50% and 5% of the duodenum were 14.5 Gy and 22.5 Gy, respectively. In the 12-week follow-up, there were 6 patients with grade 2 abdominal pain and diarrhea, and none with grade 3 or higher toxicities [11].

In a retrospective study of 77 patients treated at the same institution, single fraction SBRT with 25 Gy in 1 fraction of the pancreas again demonstrated effective local control, though there was a higher risk of toxicity during the longer follow-up of 12 months. It is important to recognize that 21% of patients in the study also received 45–54 Gy of fractionated radiotherapy prior to SBRT, leading to increased toxicity. In the 12-month follow-up, the freedom from local progression (FFLP) was 84%, and the progression-free survival was 9%. The isolated local failure rate was only 5% at 6 months and 12 months. The overall survival was 56% at six months and 21% at 12 months, and the median survival in patients with locally advanced disease was 6.7 months following treatment. Four patients (5%) experienced acute grade ≥ 2 toxicity. Three patients (4%) had late grade 2 toxicity, and seven patients (9%) had late grade ≥ 3 toxicity. Of these patients, 1 of the 4 acute toxicities and 3 of the 10 late grade ≥ 2 toxicities had received previous external beam fractionated radiation [12].

Another prospective study of 16 patients investigating 25 Gy in 1 fraction delivered between cycles 1 and 2 of gemcitabine had similarly high rates of local control. FFLP was 100% at 1 year. Three patients (19%) developed local recurrences during the median follow-up of 22.3 months. The median overall survival was 11.4 months. However, there was a significant risk of late GI toxicity. Three patients (19%) had acute pain and gastritis, one of which required J-tube placement for grade 3 gastric outlet obstruction. Seven patients (47%) had late grade ≥ 2 toxicities including gastric or duodenal ulcers or duodenal perforation. The average duodenal volume irradiated to ≥22.5 Gy (95% prescribed dose), ≥18.75 Gy (75% prescribed dose), and ≥12.5 Gy (50% prescribed dose) was higher in the patients who developed ulcers but did not reach statistical significance [13].

## 5. Multi-Fraction Stereotactic Body Radiation Therapy

In an effort to decrease toxicity, multi-fraction regimens were explored in a number of studies. A three fraction SBRT approach followed by gemcitabine was evaluated at Harvard in a retrospective study of 36 patients with locally advanced pancreatic cancer. Patients received doses ranging from 24 to 36 Gy in three fractions depending on the tumor proximity to the duodenum and stomach. At a median follow-up of 2 years, the local control rate was 78%, and the median overall survival was 14.3 months. No differences in outcomes were detected between the three dose groups, however, each group had small numbers of patients. Three patients (8%) had acute grade 3 toxicities, including two patients hospitalized with cramping, vomiting, and dehydration. Two patients had late GI bleeding [14].

In a comparison between five fraction and one fraction SBRT, a five-fraction regimen was shown to reduce GI toxicity while maintaining similar local control in unresectable disease. Patients in the five-fraction arm were treated with a dose between 25–45 Gy with a median dose of 33 Gy. Patients in the single fraction arm were treated to 25 Gy. The majority of patients also received neoadjuvant or adjuvant chemotherapy. There were no significant differences in the cumulative incidence rate of local recurrence or survival rates between single fraction and five fraction SBRT. The local recurrence rate of single fraction and five fraction SBRT at 12 months was 9.5% and 11.7% respectively, and the 12-month survival rate was 30.8% and 34.9%, respectively. Although rates of local control and survival were similar between groups, there were significantly more toxicities in the single fraction group. A total of 8 of 91 patients in the multi-fraction group (8.8%) and 19 of 76 patients in the single fraction group (25%) experienced ≥2 GI toxicities. Biologically effective dose (BED) was a significant predictor for grade ≥ 2 toxicity. Tumor location and receipt of chemotherapy were not predictive of toxicity. The rate of grade ≥ 3 gastrointestinal toxicity at 12 months was 12.3% in the single fraction group and 5.6% in the multi-fraction group, however, this was not statistically significant. Most grade 3–4 toxicities were duodenal perforation or duodenal/gastric ulcer. The dose constraints for five fraction SBRT included the volume receiving over 33 Gy (V33) was ≤1 cc for the duodenum, while the volume receiving 20 Gy (V20) was ≤3 cc for the stomach, and similarly the V15 was ≤9 cc for the bowel [15].

Another single institution retrospective review of five fraction SBRT showed similar local control rates and GI toxicities in unresectable disease. This study included 57 borderline resectable patients and 16 unresectable patients. Patients all received induction chemotherapy followed by SBRT. The region of vessel involvement was treated to a median dose of 35 Gy while the remainder of the PTV received 25–30 Gy. The local control rate for all unresected patients was 81% at 12 months and 60% at 18 months. No acute grade ≥ 3 toxicities were observed. Four patients (5.3%) had late grade 3 toxicity, with three of the four experiencing GI-related toxicity. One of the patients with GI-related toxicity had local tumor progression into the duodenum after SBRT. The authors concluded that the GI bleeding may have been related to tumor progression, radiation treatment, or both. The median volumes of duodenum receiving >30 Gy and 35 Gy were 5.2 cc and 0 cc, respectively [16].

A prospective multi-institutional study treating patients with SBRT to 33 Gy in 5 fractions along with gemcitabine before and after SBRT demonstrated low rates of acute and late GI toxicities. Acute grade ≥ 2 GI toxicity was 2% and late grade ≥ 2 GI toxicity was 11%. 1-year FFLP was only 78%; the median overall survival was 13.9 months [17].

Overall, early pancreas SBRT experiences reported significant toxicities to the adjacent bowel, most commonly the stomach and duodenum, including GI ulceration or perforation. Later SBRT studies using doses 24–36 Gy in 3–5 fractions in an effort to decrease toxicity have had effective but modest outcomes, with worsening local control and overall survival after one year of follow-up [18,19]. Multi-fraction SBRT enables conformal and well-tolerated radiation, yet the lower biologically effective dose may have led to survival outcomes similar to conventional fractionation.

## 6. Dose Escalation

Several studies have shown that increased BED may improve local control and overall survival for inoperable patients. Nearly all other disease sites treated definitively with radiotherapy utilize significantly higher doses than those reported in multi-fraction SBRT trials for pancreas cancer. With stereotactic techniques and the emergence of image guidance, radiation dose escalation can be attempted with a planning strategy including maximized target volume coverage with an ablative dose, intentional dose heterogeneity with “hotspots” in the center of the tumor, and restricted dose to the areas nearest GI organs at risk to meet dose constraints [20].

One of the first studies utilizing these techniques with intensity-modulated radiotherapy and image guidance was a retrospective cohort study by Krishnan et al. This was from MD Anderson of locally advanced pancreas cancer including patients treated with FOLFIRINOX or gemcitabine-based induction chemotherapy, which was followed by radiotherapy with concurrent gemcitabine or capecitabine-based chemotherapy. A simultaneous in-field boost (SIB) technique was utilized allowing for a higher dose to a smaller central volume in the target. Daily inspiration breath-hold respiratory gating and CT-on-Rails image registration without fiducials were used for motion management. Patients were treated with either dose escalated regimens with BED_10_ (BED using an α/β of 10) of > 70 Gy, or standard fractionation with a BED_10_ ≤ 70 Gy. In the higher dose group, patients were treated with a variety of fractionation regimens. Most in this group were treated with 63 or 70 Gy in 28 fractions, or 51.3–70.4 Gy in 13–39 fractions. Of the patients receiving dose escalated radiation, 41 patients were treated with intensity modulated radiation therapy (IMRT), and 5 patients were treated with 3D-CRT followed by an IMRT boost. Of the patients receiving standard dose radiation, 140 patients were treated with 3D-CRT and 13 were treated with IMRT.

The patients who received BED > 70 had a significantly improved overall survival of 17.8 months, compared to 15.0 months in the lower dose group. Estimated overall survival rates were 36% versus 19% at two years, and 31% versus 9% at three years. The median local regional recurrence-free survival was also longer in the high dose group (10.2 vs. 6.2 months). A total of 28% of all patients had acute grade 2 toxicities, and only one patient (2%) had grade 3 diarrhea. Four patients (13%) required blood transfusions for anemia, and one patient had a GI bleed. Toxicities were similar between high and low dose groups. However, dose escalation was only used for tumors more than 1 cm away from GI structures [21].

A subsequent cohort study at the Memorial Sloan Kettering treated patients with hypo-fractionated ablative radiotherapy with BED_10_ beyond 97 Gy. Tumors less than 1 cm from the stomach or bowel were treated with 75 Gy in 25 fractions (BED_10_ = 97.5) and tumors 1 cm or further from the stomach or bowel were treated with 67.5 Gy in 15 fractions (BED_10_ = 97.88). Respiratory motion management and daily cone beam computed tomography (CT) were utilized. Most patients also received induction chemotherapy. The median overall survival from diagnosis was 26.8 months, and overall survival from radiotherapy was 18.4 months. The 1-year and 2-year overall survival from radiation was 74% and 38%, respectively. The 1-year and 2-year cumulative incidence of locoregional failure was 17.6% and 32.8%, respectively. Of the 119 patients, 16 (13.4%) had grade 3 GI toxicities, including 10 patients with upper GI bleeding. There was no grade 4 of 5 toxicities [22].

Zhu et al. reviewed patterns of local failure in medically inoperable patients treated with SBRT and chemotherapy and illustrated the benefit of a higher BED. The median BED_10_ was 64.35 Gy delivered in 5–8 fractions. A prescription dose with BED_10_ of ≥60 Gy was significantly correlated with higher local control and improved overall survival. Additionally, patients with BED_10_ of ≥60 Gy had fewer isolated in-field recurrences, as well as fewer in-field plus outside-the-field and distant recurrences. In tumors prescribed a regimen with BED_10_ < 60 Gy, portions of the tumor could still receive BED_10_ of 60 Gy if there were “hotspots” within the tumor volume. The authors reevaluated failures within the BED_10_ = 60 Gy isodose line in these patients to evaluate whether there were fewer recurrences in the region receiving at least this BED. Patterns of recurrence within the BED_10_ = 60 Gy isodose line in the BED_10_ < 60 Gy group were similar to in-field recurrences in the group prescribed BED_10_ ≥ 60 [23].

## 7. Standard CT Guided Radiotherapy

The SBRT and dose escalation studies described thus far treated patients with traditional CT-guided radiotherapy (CTgRT). CTgRT requires motion management techniques, including 4-dimensional CT (4DCT) and image guidance with cone beam CT (CBCT). The respiratory cycle as well as bowel filling and emptying cause significant motion of the pancreas and other abdominal organs. However, the poor soft tissue delineation of CT imaging limits the accuracy of determining treatment volumes. Radio-opaque fiducial markers are utilized to better localize the tumor during simulation as well as prior to each fraction on CBCT. However, even with fiducial markers, 4DCT at simulation may not adequately account for intrafraction tumor motion [24]. Additionally, use of 4DCT to create an internal target volume (ITV) or a larger PTV encompassing the range of tumor motion also increases the risk of high dose to nearby OARs and thus toxicity to the patient.

CT based real-time target tracking with kV imaging on a conventional linear accelerator has been explored. A single institution study from the University of Colorado describes tracking with kilovoltage (kV) images approximately every 5–6 s, either every 20 degrees of volumetric arc therapy (VMAT) delivery for patients with abdominal compression, or at the start of each “beam on” cycle for patients with respiratory gating. In order to account for deviations in the tumor location, the treatment table could be shifted to realign the tumor. The gating threshold could also be adjusted, or treatment halted, if there were changes in the patient’s breathing. The median treatment time, even with treatment pauses, was 8.1 min. Using dosimetric simulations, only about 45% of shifts resulted in improved target coverage [25].

The major benefit of CTgRT is the short treatment time, which may help limit intra-fraction tumor and OAR motion and increase patient convenience. However, the advances in motion management including image guidance and respiratory gating do not account for inter- and intra-fraction motion of GI organs. MR-guided radiotherapy (MRgRT) with adaptive planning provides a solution to several limitations of CTgRT including optimized soft tissue delineation as well as tumor and OAR tracking.

## 8. MRgRT vs. CTgRT

The proximity of OARs is an obstacle to the goal of dose escalation in the treatment of inoperable pancreatic cancers [26]. Stereotactic magnetic resonance (MR) guided adaptive radiation therapy (SMART) is a technique that allows the delivery of higher doses to the gross disease in the pancreas while sparing normal tissues. The process of treatment on an MR linear accelerator (LINAC) includes re-contouring OARs near the GTV based on the MRI appearance on the day of treatment. A previously developed plan, called the predicted plan, is fused to assist with decision-making [27]. The predicted plan uses the baseline simulation plan and is compared with the new MRI images with adjusted contours. Plan re-optimization is performed to meet dose constraints to the OARs and improve target volume coverage as needed. Gating is accomplished with the use of a biofeedback monitor that shows the patient their real-time sagittal cine MR to demonstrate their breath-hold positions. The therapists provide coaching in order to facilitate this process and decrease the treatment times.

There are several benefits to MRgRT over CTgRT, including better soft tissue contrast, real-time delineation of structures such as the pancreas and nearby organs at risk, treatment gating based on tumor tracking, and daily adaptive re-planning to account for intra- and inter-fraction changes in treatment setup or patient anatomy.

In general, CT images are best at distinguishing structures with very different Hounsfield units, such as between tissue, air, and bone. Adjacent tissue structures with similar X-ray attenuation properties are difficult to discriminate unless there is a visible interface between structures. In contrast, MRI offers better characterization of soft tissues even with similar Hounsfield units or electron densities [28]. The superior visualization of pancreas tumors obviates the need for fiducial placement, preventing delays in initiation of radiotherapy. This also avoids the small risk of complications with fiducial placement, including pancreatitis [29]. Rarely, fiducial markers can migrate, which would compromise targeting of radiation [30].

**Figure 1:**
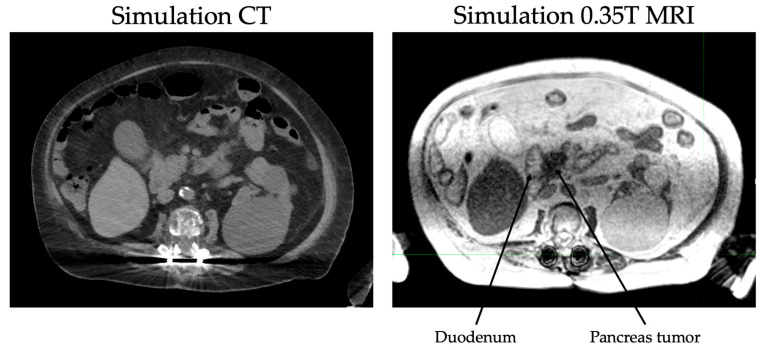
CT vs. MRI at simulation for a patient with pancreas head adenocarcinoma.

The continuous tracking of tumor volume during treatment also eliminates the need for an ITV or expanded PTV and thus reduces the dose to normal tissue. In pancreatic cancers, daily MR guidance is especially helpful for delineation of the stomach and duodenum from the pancreas (Figure 1). Immobilization devices are not needed due to real time tracking of inter- and intra-fraction motion. Due to superior tracking of both targets and OARs, ablative doses can be prescribed to tumors regardless of proximity to GI structures as compared to CT-based radiotherapy. For example, dose-escalation studies such as Krishnan et al. [21] limited doses to tumors within 1 cm of GI structures due to toxicity concerns. With MRgRT, limitation of the prescription dose is not necessary. Online adaptive planning allows recontouring of relevant structures and recalculation of the dose received by the tumor and OARs with each fraction [31,32,33].

### Dosimetric Benefit of MRgRT

A recent retrospective analysis from Munich described the dosimetric benefits of MRgRT across multiple tumor sites including the pancreas. Treatment plans were adapted in 92% of fractions in the pancreas group. Of the tumor entities evaluated, the pancreas subgroup demonstrated the largest median dose reduction (~87.0%) to OARs close to the PTV in the adapted plans. The most common OARs considered in adaptive planning for these patients were duodenum, stomach, and bowel. The volume receiving 33 Gy (V33_Gy_) for the duodenum, stomach, and bowel had a median reduction of 97.1%, 99.1%, and 98.6% respectively.

While the dose to OARs was significantly decreased, the dose to the target volumes either improved or did not significantly change. The median dose to 95% and 98% of the PTV was significantly increased by 5.7% and 11.0%, respectively, while the dose to the GTV did not exhibit significant differences in the adapted plans.

Compared to other tumor sites, plan adaptation was primarily used in pancreas patients to reduce doses to OARs. The GTV coverage was sufficient in most cases but not improved with plan adaptation. In several cases, OAR sparing was prioritized over GTV coverage. The high percentage of adapted fractions and subsequent dose reduction to OARs demonstrates the utility of online adaptation in pancreas cancers [34].

## 9. SMART Efficacy and Reduction of Toxicity

The first-generation SMART utilized cobalt-60 and achieved good local control comparable to the previously achieved best results without high-grade toxicity in patients receiving dose escalation. The first MR-based LINAC began treating patients in 2017 using intensity modulated radiation therapy instead of cobalt-60. This increased the potential for higher doses than first-generation machines due to the greater dose conformality.

Dose escalation using SMART has been shown to improve local control and overall survival. A retrospective multi-institutional study using MR-guided radiation therapy stratified inoperable patients receiving conventional fractionation, hypofractionation, and SBRT into high dose (BED_10_ > 70) and standard dose (BED_10_ ≤ 70) groups. A variety of induction chemotherapy regimens were used, and concurrent chemotherapy was given with conventional fractionated and hypo-fractionated radiation treatments. High dose patients had a statistically significant improvement in overall survival, 49% versus 30%, compared to standard dose patients at two years. The high dose group had a median overall survival of 20.8 months. The median overall survival of 10.8 months from the start of radiation in the standard dose cohort was similar to historical controls receiving conventional chemoradiation, indicating that MRgRT without dose escalation may not improve outcomes. Freedom from distant failure between the two groups was similar despite the large overall survival difference. The authors hypothesized that ablative doses of radiation may have prevented symptoms caused by local failure such as anorexia and nausea that often prevent patients from receiving effective systemic therapy at progression. There were three patients with grade ≥ 3 GI toxicity in the standard dose group, but none in the high dose group, suggesting that plan adaptation and decreased concurrent chemotherapy contributed to a lower risk of toxicity [35].

A single institution retrospective study was the first report of SMART with 50 Gy in 5 fractions (BED_10_ = 100 Gy) for unresectable pancreatic cancer. This ablative dose was prescribed to all tumor geographies including tumors abutting nearby OARs. A total of 80% of patients had tumors abutting an OAR. Additionally, five patients had tumors invasion into a GI endoluminal structure, which is traditionally a contraindication to SBRT. Patients were treated using either the MRIdian Cobalt-60 system or an MR LINAC system. Forty-four patients were treated between 2014 and 2019. Despite the proximity of tumors to OAR in many patients, there were low rates of GI toxicities. Late toxicity was limited to two patients (4.6%) with grade 3 toxicities and three patients (6.8%) with grade 2 toxicities, including GI ulcer or perforation and gastric bleeding. Patients had a median survival of 15.7 months, and overall survival was 68.2% at one year and 37.9% at two years. Local control at one year was excellent at 84.3%. Furthermore, there was a median treatment-free interval of 7 months, and several patients had a treatment break of 1 year or longer. This treatment-free interval creates the possibility that SMART may also be helpful in frail patients unable to tolerate long courses of high-dose chemotherapy [36].

Chuong et al. was the first to report of five fractions ablative SMART for inoperable pancreatic cancer exclusively on an MR LINAC. This retrospective analysis of 35 patients, most of whom had locally advanced disease and received induction chemotherapy, included patients treated to a median dose of 50 Gy in 5 fractions. Patients were treated with mid-inspiration breath hold. Any portion of the PTV overlapping the planning organ at risk volumes (PRV) were limited to 35 Gy in order to meet dose constraints. The remainder of the PTV was prescribed 50 Gy, though several patients early in the study were prescribed 40 Gy due to concerns about dose tolerance. The hotspot was optimized to be ≥120–130% of the prescription dose and encompassed as much of the PTV not overlapping PRV as possible. The institution initially treated gross disease only. Elective nodal irradiation (ENI) was later added as the treatment was observed to be well-tolerated because it was thought that ENI may reduce locoregional recurrence [37]. Though this remains unproven, ENI was delivered to over half of patients.

During the median follow-up of 10.3 months, acute grade 3 toxicity and late grade 3 toxicity were both 2.9%. One-year progression-free survival was 52.4% and overall survival was 58.9%, though almost half of patient deaths were unrelated to pancreatic cancer. One-year local control was 87.8%, and only one patient had progression within the PTV [38].

## 10. Challenges of SMART

There are several challenges associated with SMART. MR simulation and treatments are more time intensive than CT simulation and treatments. During each fraction of radiation delivery, adaptive techniques require longer on-table time for patients. The Phase I SMART trial assessing feasibility of SMART for abdominal malignancies was not able to meet its primary endpoint of delivery of treatment within 80 min in >75% of cases. An endpoint of 80 min was chosen by the study physician’s prediction of maximum tolerated treatment time for patients. Only about 57% of fractions were completed in less than 80 min, though >75% of fractions were completed within 90 min [39].

Adaptive planning is not only time intensive, but also resource intensive. Most members of the radiation treatment team are trained to use CT-based systems. Departments that adopt SMART must invest significant time and resources into training radiation therapists, nurses, dosimetrists, physicists, and physicians. This additional training includes proper screening of patients for MRI candidacy, as MRI has several contraindications not applicable to CT. These include the presence of implanted medical devices and metal fragments in patients. The strong magnetic fields of the MRI attract ferromagnetic objects which can become airborne projectiles. MRI can cause interference with certain implanted cardiac devices like pacemakers and cause life-threatening arrhythmias [40]. All patients and staff must be scanned for magnetic objects prior to entering the MRI room. Patients with severe claustrophobia also may have difficulty with MRgRT.

## 11. Conclusions

Overall, adaptive SBRT with real-time MR guidance (SMART) offers enhances dose coverage and optimal sparing of normal tissues for treatment of pancreatic cancer compared to traditional CT-based SBRT. The literature thus far shows promising local control and overall survival outcomes as well as improvement in gastrointestinal toxicities (Table 1).

The role of radiotherapy in pancreas cancer has evolved from standard fractionation with disappointing outcomes, to single fraction SBRT with significant toxicity and now to multifractionated MR-guided adaptive SBRT. Available data from retrospective studies demonstrate that dose escalation using SMART can significantly improve progression-free survival in a disease with limited overall survival, while maintaining the acceptable risk of GI toxicity. Based on this, there is now an ongoing prospective phase II trial evaluating grade 3 or greater GI toxicity after MRI-guided adaptive radiotherapy using 50 Gy in 5 fractions for patients with borderline resectable or inoperable pancreatic cancer [41].

Moreover, MR-guided adaptive radiotherapy prescribed to high BEDs can potentially provide patients a significant treatment-free intervals. Further studies are needed, including the ongoing phase II SMART trial, which will provide more prospective data on the utility of MR-based adaptive planning.

## Figures and Tables

**Table 1:** Representative Modern Pancreas Cancer Radiotherapy Trials.

Study	N *	RT ** Dose	RT Technique	Median Overall Survival (Months)	Local Control	GI toxicity (gr ≥ 2)
Chauffert et al. [8]	119	60 Gy/30 fx + 5Fu + cis → gem, vs. gem	3D-CRT conventional fractionation	8.6 vs. 13 months, *p* = 0.03		Acute ≥ gr 3 65.5% versus 40%; *p* = 0.008 induction phase, 78% versus 40%; *p* = 0.0001 maintenance phase (includes non-GI)
ECOG 4201 [9]	74	50.4 Gy/28 fx → gem, vs. gem	3D-CRT conventional fractionation	11.1 vs. 9.2 months, *p* = 0.017		Acute ≥ gr 3 82% vs. 80% (includes non-GI)
LAP-07 [10]	269	Gem ± erlotinib → 54 Gy/30 fx + cape vs. gem alone	3D-CRT conventional fractionation	15.2 vs. 16.5 months, NS	Locoregional 68% vs. 54%, *p* = 0.03	Acute ≥ gr 3 23.1% vs. 19.8% (non-heme)
Koong et al. [11]	15	15–25 Gy/1 fx	SBRT	8 (25 Gy group)	12 wks 100% (25 Gy group)	Acute 40%
Chang et al. [12]	77	25 Gy/1 fx	SBRT	11.9	1 y 84%	Acute 5% Late 13%
Schellenberg et al. [13]	16	25 Gy/1 fx	SBRT	11.4	1 y 100%	Acute 19% Late 47%
Mahadevan et al. [14]	36	24–36 Gy/3 fx	SBRT	14.3	2 y 78%	Acute 41% Late 5%
Pollom et al. [15]	167	25 Gy/1 fx vs. 25–45 Gy/5 fx	SBRT	13.6	1 y 90.5% vs. 88.3%, NS	25% vs. 9%
Chuong et al. [16]	73	35–50 Gy/5 fx	SBRT	15 (LAPC)	1 y 81% (nonsurgical)	Late ≥ gr 3 5.3%
Herman et al. [17]	49	33 Gy/5 fx	SBRT	13.9	1 y 78%	Acute 2% Late 11%
Krishnan et al. [21]	200	50–70.4 Gy/ 5–39 fx	3D-CRT and IMRT; conventional fractionation and hypofractionation	17.8 vs. 15 mo, *p* = 0.03 *	1 y locoregional control 21% vs. 9% *	Acute 30% (high dose group, includes non-GI)
Reyngold et al. [22]	119	67.5–75 Gy/ 15–25 fx	Hypofractionated	26.8 (from diagnosis), 18.4 (from RT)	1 y locoregional control 82.4%	Late ≥ grade 3 13.4%
Rudra et al. [35]	44	30–67.5 Gy/ 5–28 fx	MRgRT; conventional fractionation, hypofractionation, SBRT	20.8 vs. 10.8 ***	2 y 77% vs. 57%, *p* = 0.15 ***	Acute ≥ grade 3 7% vs. 0% ***
Hassanzadeh et al. [36]	44	50 Gy/5 fx	MRgRT SBRT	15.7	1 yr 84.3%	Late 11.3%
Chuong et al. [38]	35	40–50 Gy/5 fx	MRgRT SBRT	9.8	1 yr 87.8%	Late 5.7%

* N = number of patients. ** RT = radiation therapy. *** BED > 70 vs. BED ≤ 70 groups.
